# The hOGG1 Ser326Cys gene polymorphism and susceptibility for bladder cancer: a meta-analysis

**DOI:** 10.1590/S1677-5538.IBJU.2015.0446

**Published:** 2016

**Authors:** Cao Wenjuan, Lu Jianzhong, Li Chong, Gao Yanjun, Lu Keqing, Wang Hanzhang, Wang Zhiping

**Affiliations:** 1 Institute of Urology, The Second Hospital of Lanzhou University, Key Laboratory of Urological Diseases in Gansu Province, Gansu Nephro - Urological Clinical Center, Lanzhou, China; 2 Department of Urology, University of Texas Health Science Center San Antonio, San Antonio, Texas, USA

**Keywords:** oxoguanine glycosylase 1, human [Supplementary Concept], Polymorphism, Genetic, Urinary Bladder Neoplasms, Meta-Analysis as Topic

## Abstract

**Objective:**

To assess the susceptibility of the hOGG1 genetic polymorphism for bladder cancer and evaluate the impact of smoking exposure.

**Materials and Methods:**

Articles included in PubMed, Medline and Springer databases were retrieved using the following key words: “human 8-oxoguanine DNA glycosylase”, “OGG”, “OGG1”, “hOGG1”, “genetic variation”, “polymorphism” , “bladder cancer”, and “bladder carcinoma” to Meta-analysis was performed to detect whether there were differences between the bladder cancer group and the control group about the distribution of genotypes of the hOGG1 gene.

**Results:**

The results showed that there are no significant associations between the hOGG1 326Cys polymorphism and bladder cancer: GG vs. CC (OR: 1.09, 95% CI: 0.85–1.40, p=0.480); GC vs. CC (OR: 1.05, 95% CI: 0.85–1.28, p=0.662); GG+GC vs. CC (OR: 1.04, 95% CI: 0.89–1.21, p=0.619); GG vs. GC+CC(OR: 1.02, 95% CI: 0.78–1.33, p=0.888); G vs. C (OR: 1.01, 95% CI: 0.91–1.13, p=0.818). In the smoker population, no significant associations between the hOGG1 326Cys polymorphism and bladder cancer were observed for all the models. However, individuals carrying the hOGG1 Cys326Cys genotype have increased risk for bladder cancer compared to those carrying the hOGG1 Ser326Ser genotype in the non-smoker Asian population.

**Conclusion:**

The hOGG1 326Cys polymorphisms aren't a risk factor for bladder cancer, especially in the smoker population. But GG genotype is a risk factor for bladder cancer to the non-smoker Asian population compared with CC genotype.

## INTRODUCTION

Bladder cancer is the most common malignancy of the urinary system over the world (1). It had been recognized that the occurrence of bladder cancer results from a series of external environment, genes mutation and the interaction of genes with the environment (2, 3). Existing epidemiological findings suggest that smoking and occupational exposure to carcinogens are the most important external hazard factors for bladder cancer (4, 5). However, bladder cancer pathogenesis is still unclear. DNA experience oxidative damage in the role of internal and external environment, which can initiate different repair pathways in cells (6, 7). In recent years, many scholars explored the mechanism of bladder cancer according to the susceptibility of DNA repair gene polymorphism for the occurrence of bladder cancer (8–10). DNA repair gene single nucleotide polymorphisms (single nucleotide polymorphism, SNP) will change the DNA repair capacity of patient, and further increase genomic instability and invasiveness of tumor cells.

The human 8-oxoguanine DNA glycosylase (hOGG1) gene is located on human chromosome 3p26.2 (11), major repairing damage caused by 8-bird Purine (8-OH-dG) and closing to cancer occurrence and development (12). The hOGG1 repair gene is necessary in effective base excision repair (BER) pathway (11). Because of the characteristics of SNP, the interaction between gene mutation and environment could lead to DNA repair defects, and then increasing incidence of certain tumors (13). In different populations, hOGG1 genetic polymorphism is observed. The C/G polymorphism at 1245bp (C1245G) in exon 7 of the hOGG1 gene results in an amino acid substitution of serine (Ser) with cysteine (Cys) at codon 326. The results of studies about the relationship between hOGG1 genetic polymorphism and bladder cancer aren't uniform and previous meta-analysis showed there are no significant association between hOGG1 genetic polymorphism and bladder cancer (14–16). But in recent years, many researches had indicated that the hOGG1 Cys326Cys is a susceptibility gene for the occurrence of bladder cancer and smoking status plays an important role in their relationship (17, 18). So, we implemented a meta-analysis including the latest research paper to reveal the role of hOGG1 genetic polymorphism in the occurrence of bladder cancer and explain the possible causes.

## MATERIALS AND METHODS

### Publication search eligibility of relevant studies

All the case-control studies were found out by researching the database of PubMed, Medline and Springer databases using the following key words: “human 8-oxoguanine DNA glycosylase”, “OGG”, “OGG1”, “hOGG1”, “genetic variation”, “polymorphism”,“bladder cancer”, and “bladder carcinoma”. Human-associated studies were concluded, and no language constraints. In addition, references of retrieved publications were searched manually. If the data were missing, we communicated with the author by e-mail. The most recent or complete articles with the largest number of subjects were selected from the overlapping data by the same authors. The last search was updated on 2014. Studies included meet the following criteria: (1) an independent case-control trial, (2) evaluation of the hOGG1 Ser326Cys gene polymorphisms and susceptibility for bladder cancer and (3) contain available genotype frequency. Those without controls and duplicate of previous publication were excluded.

### Data extraction

Two investigators independently screened documents and extracted data, and then checked the results of the included studies. In the present study, we sought the following information from each publication: the first author's last name, year of studies, country of origin, ethnicity, source of controls (population or hospital-based controls), genotyping method and number of genotyped cases and controls. We used the Newcastle-Ottawa Scale (NOS) to assess the quality of the studies included in our analysis. Different ethnic groups were categorized as Caucasian and Asian to assess the effect of hOGG1 Ser326Cys polymorphism. Gene-by-environment interaction analyses were assessed according to smoker exposure (smoker or non-smoker).

### Statistical analysis

The intensity of the associations between hOGG1 Ser326Cys polymorphisms and bladder risk was measured by odds ratios (ORs) with 95% confidence intervals (CIs). For hOGG1 Ser326Cys polymorphism, the risk of the dominant model (GG+GC vs. CC), the recessive model (GG vs. GC+CC), the codominant model (GC vs. CC; GG vs. CC), and the allele model (G vs. C) were evaluated respectively. Subgroup analyses were also performed by ethnicity and smoker exposure. Hardy–Weinberg equilibrium (HWE) was used to assess the genotype frequency of hOGG1 Ser326Cys polymorphisms among the controls by χ2 test. Meta-analysis's process references Cochrane organization guidelines, using Q statistics to analyze the heterogeneity between the trials, and a P value of <0.10 was considered significant. The fixed-effects model and the random-effects model were based on the Mantel-Haenszel method, using random-effects models when I^2^ is >50%, and fixed-effects models when I^2^ is <50%. Funnel plots was applied to test the publication bias. All analyses were done with Stata software 12.0 using two-sided P values.

## RESULTS

### Characteristics of studies

A total of 10 articles related to the search words and complied with the present inclusion criteria, including 4319 cases and 4716 controls. All studies were case-control studies, in which there were five studies of Asian descendents, five studies of Caucasian descendents. A classic polymerase chain reaction–restriction fragment length polymorphism (PCR–RFLP) assay, TaqMan assay and amplification refractory mutation specific polymerase chain reaction (ARMS-PCR) assay were conducted in six, three and one of the ten studies, respectively. In most studies, bladder cancer was diagnosed histologically or pathologically. Controls were mainly matched for sex and age, of which one were population based and nine were hospital based. The characteristics of selected studies are summarized in Table-1 (10, 16–24). Detailed genotype frequency data were reported in all studies and the allele frequency were also calculated. One study genotype distributions among the controls of all studies were deviated from the HWE, and the total controls were not consistent with HWE. The information of genotype, allele frequencies and HWE are shown in Table-2.

**Table 1:** Characteristics of studies included in the meta-analysis.

First author [reference]	Country/Region	Ethnicity	Genotyping method	Cases (age)	Controls (age)	Design of Experiment	NOS score
Kim (2005) (19)	Korea	Asian	PCR-RFLP	N=153 (62.9±11.8 years)	N=153 (60.7±11.8 years)	Hospital-based	6
Karahalil (2006) (20)	Turkey	Caucasian	PCR-RFLP	N=100 (mean age 59.87 years)	N=100 (mean age 59.33 years)	Hospital-based	6
Huang (2007) (21)	USA	Caucasian	Taqman	N=696 (63.94±11.17 years)	N=629 (62.77±10.50 years)	Hospital-based	7
Figueroa (2007) (22)	Spain	Caucasian	Taqman	N=1150 (66±10 years)	N=1149 (65±10 years)	Hospital-based	6
Arizono (2008) (23)	Japan	Asian	PCR-RFLP	N=251 (68.2±11.2 years)	N=251 (68.1±11.7 years)	Population-based	6
Narter (2009) (10)	Turkey	Caucasian	PCR-RFLP	N=83 (63.43±11.74 years)	N=45 (59.98±9.71 years)	Hospital-based	7
Wang (2011) (24)	Taiwan	Asian	PCR-RFLP	N=460 (62.7±10.9 years)	N=540 (61.9±11.0 years)	Hospital-based	7
Mittal (2011) (30)	India	Asian	ARMS-PCR	N=212 (59.6±12.4 years)	N=250 (58.8±10.8 years)	Hospital-based	6
Ma (2012) (27)	China	Asian	TaqMan	N=1050 (65±5 years)	N=1404 (65±5 years)	Hospital-based	6
Ramaniuk (2014) (28)	Belarus	Caucasian	PCR-RFLP	N=336 (67.0±10.7 years)	N=370 (64.5±13.5 years)	Hospital-based	8

**Table 2:** Genotype, allele frequencies and HWE of the studies.

First author	Genotype						Allele				HWE(p)
	Cases n (%)			Control n (%)			Cases n (%)		Controls n (%)		
	Ser/Ser	Ser/Cys	Cys/Cys	Ser/Ser	Ser/Cys	Cys/Cys	Ser	Cys	Ser	Cys	
Kim (2005)	37	90	26	38	70	45	164	142	146	160	0.30
Karahalil (2006)	40	47	12	62	20	18	127	71	144	56	<0.001
Huang (2007)	375	209	29	348	216	36	959	267	912	288	0.75
Figueroa (2007)	649	383	56	596	361	61	1681	495	1553	483	0.52
Arizono (2008)	61	107	83	67	135	49	229	273	269	233	0.20
Narter (2009)	37	13	8	18	18	0	87	29	54	18	0.08
Mittal (2011)	92	93	27	122	111	17	277	147	355	145	0.21
Wang (2011)	55	227	178	82	246	212	337	583	410	670	0.45
Ma (2012)	155	551	344	212	676	514	861	1239	1100	1704	0.67
Ramaniuk (2014)	223	94	18	221	132	13	540	130	574	158	0.21
Total	**1724**	**1814**	**781**	**1766**	**1985**	**965**	**5262**	**3376**	**5517**	**3915**	**<0.001**

**Figure 1:**
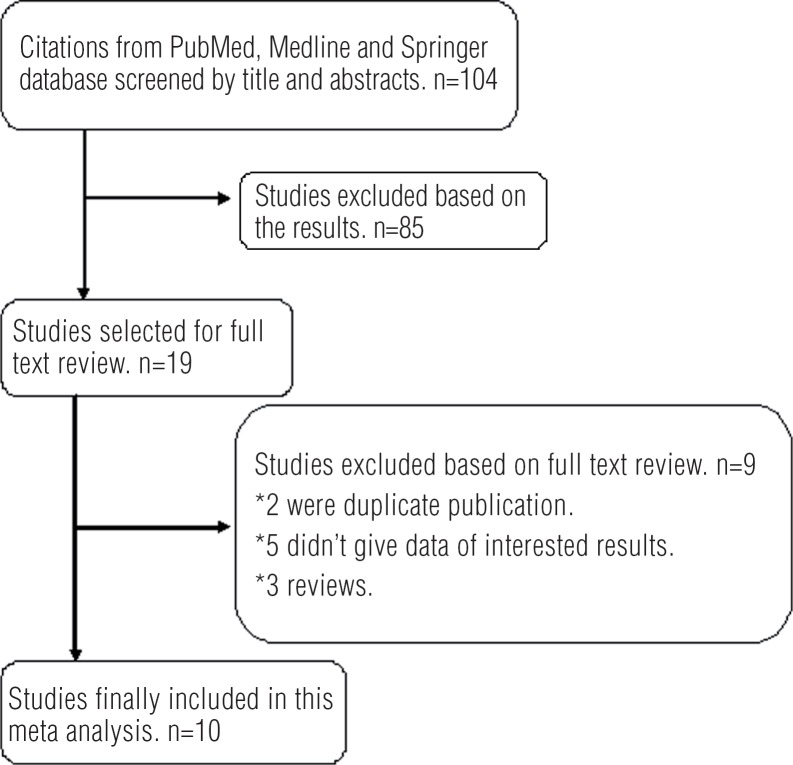
Flow chart of the study selection process.

Four of these studies investigated the interactions between hOGG1 Ser326Cys polymorphisms and smoker exposures, including 1774 smokers (927 case and 847 controls) and 1992 non-smokers (748 cases and 1224 controls). The data only showed in recessive model by the studies of Ma et al. In the gene-by-environment interaction analyses, subgroup analysis by ethnicity was also carried out. The studies about smoker exposure are shown in Table-3. The process of evaluating articles for this meta-analysis is shown in Figure-1.

**Table 3:** Genotype frequency and distribution according to smoking status.

	Author	Genotype					
		Cases			Control		
		Ser/Ser	Ser/Cys	Cys/Cys	Ser/Ser	Ser/Cys	Cys/Cys
Smoke							
	Karahalil (2006)	14	16	4	27	9	7
	Arizono (2008)	42	72	51	42	83	33
	Ma (2012)	344		153	341		195
	Ramaniuk (2014)	155	62	14	60	44	6
Non-smoke							
	Karahalil (2006)	7	4	2	38	9	10
	Arizono (2008)	19	35	32	25	52	16
	Ma (2012)	362		191	547		319
	Ramaniuk (2014)	61	31	4	142	80	6

### Main Meta-Analysis Results

**Figure 2:**
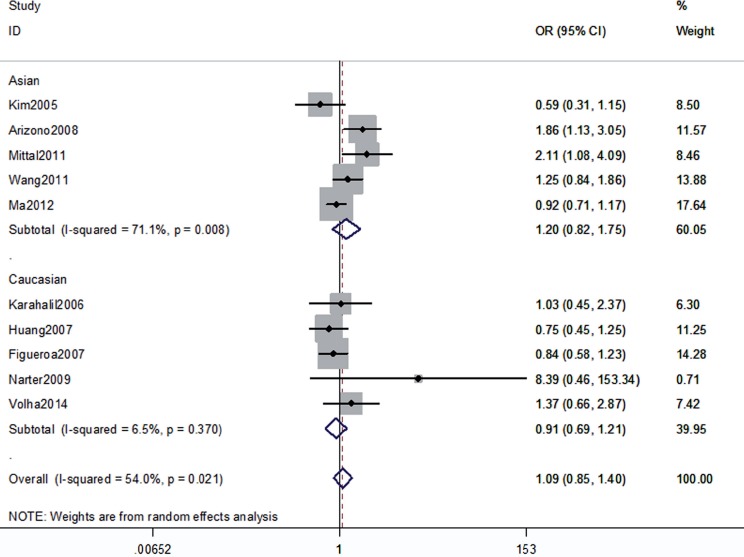
Overall meta-analysis and subgroup analysis by ethnicity for GG genotype versus CC genotype.

**Figure 3:**
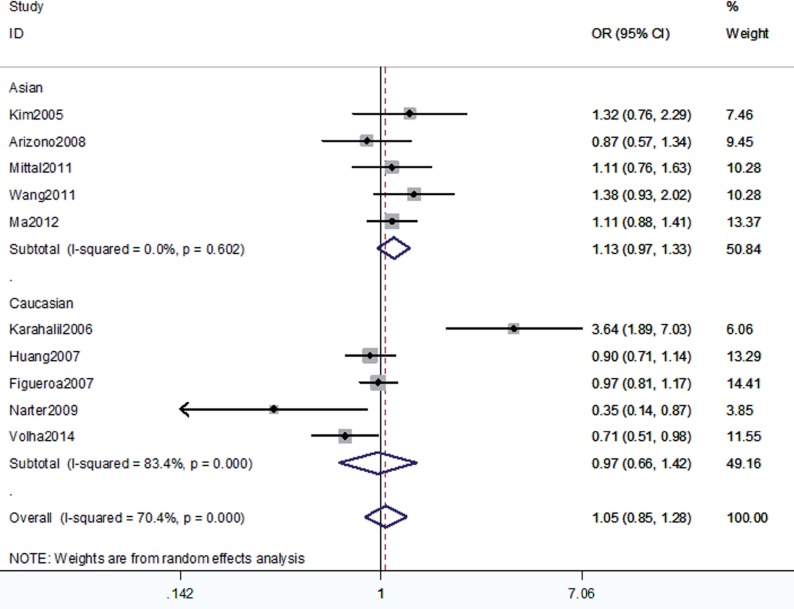
Overall meta-analysis and subgroup analysis by ethnicity for GC genotype versus CC genotype.

**Figure 4:**
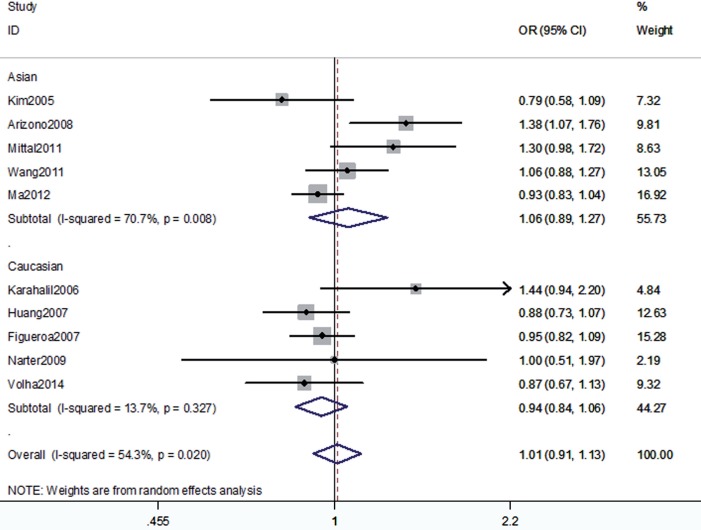
Overall meta-analysis and subgroup analysis by ethnicity for G allele versus C allele.

**Figure 5:**
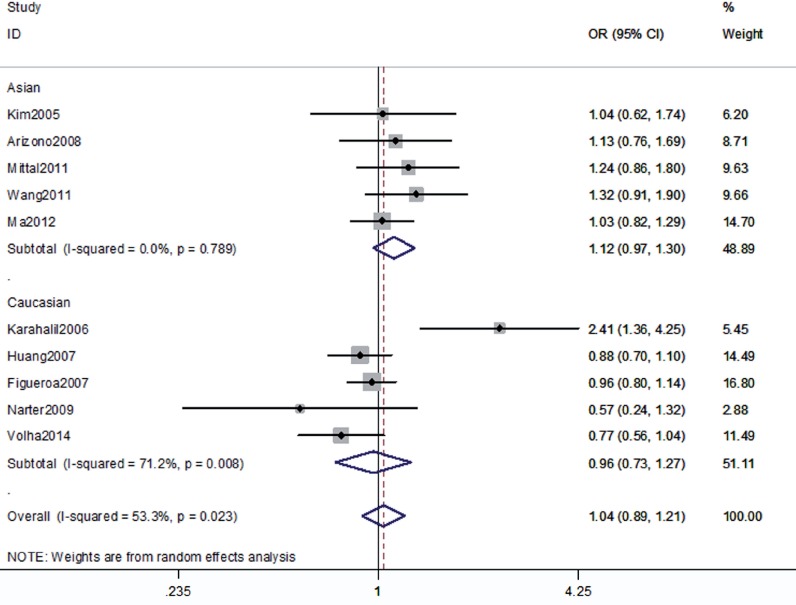
Overall meta-analysis and subgroup analysis by ethnicity for (GG+GC) genotype versus CC genotype.

**Figure 6:**
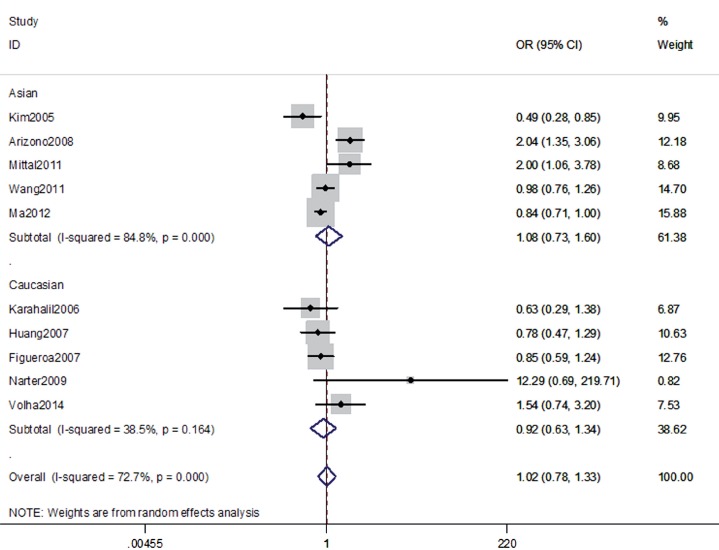
Overall meta-analysis and subgroup analysis by ethnicity for GG genotype versus (GC+CC) genotype.

The association between the hOGG1 326Cys polymorphism and bladder cancer risk is shown in Figures 2–6. In the codominant model (GG vs. CC; GC vs. CC), the dominant model (GG+GC vs. CC), the recessive model (GG vs. GC+CC), and the allele model (G vs. C), there were no significant associations between the hOGG1 326Cys polymorphism and bladder cancer: GG vs. CC (OR: 1.09, 95% CI: 0.85–1.40, p=0.480); GC vs. CC (OR: 1.05, 95% CI: 0.85–1.28, p=0.662); GG+GC vs. CC (OR: 1.04, 95% CI: 0.89–1.21, p=0.619); GG vs. GC+CC (OR: 1.02, 95% CI: 0.78–1.33, p=0.888); G vs. C (OR: 1.01, 95% CI: 0.91–1.13, p=0.818). In the subgroup analysis by ethnicity, no significant results were found for Asian and Caucasian subjects in different models.

**Figure 7:**
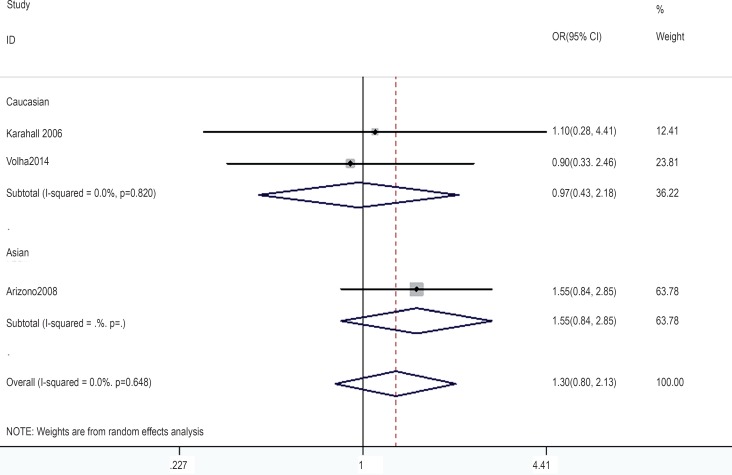
Overall meta-analysis and subgroup analysis by ethnicity for GG genotype versus CC genotype in the smoker population.

**Figure 8:**
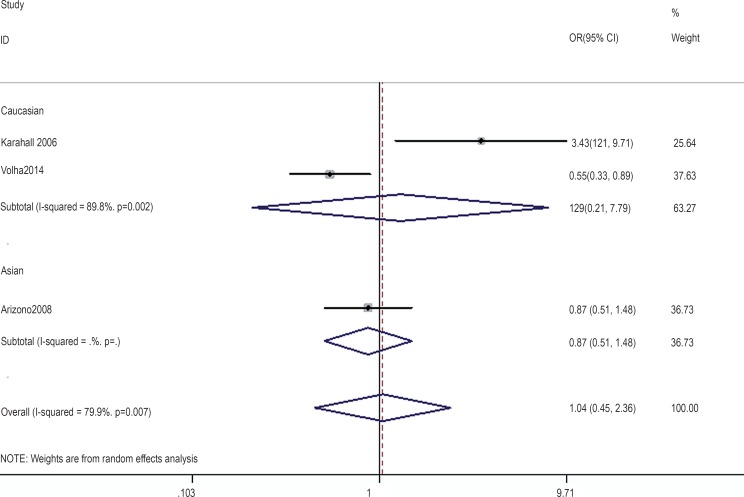
Overall meta-analysis and subgroup analysis by ethnicity for GC genotype versus CC genotype in the smoker population.

**Figure 9:**
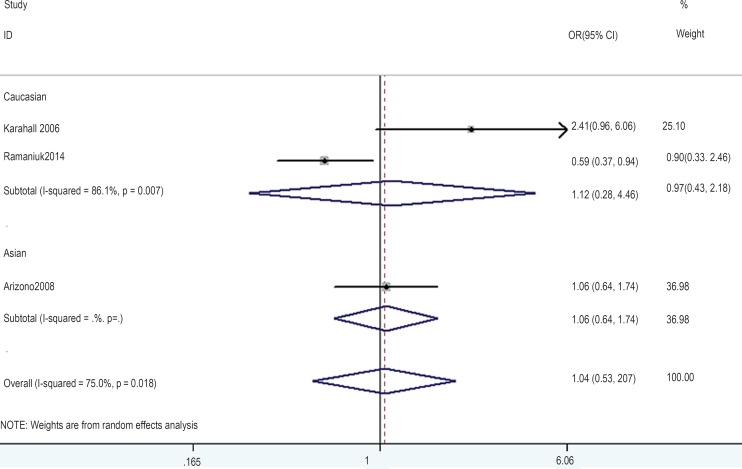
Overall meta-analysis and subgroup analysis by ethnicity for (GG+GC) genotype versus CC genotype in the smoker population.

**Figure 10:**
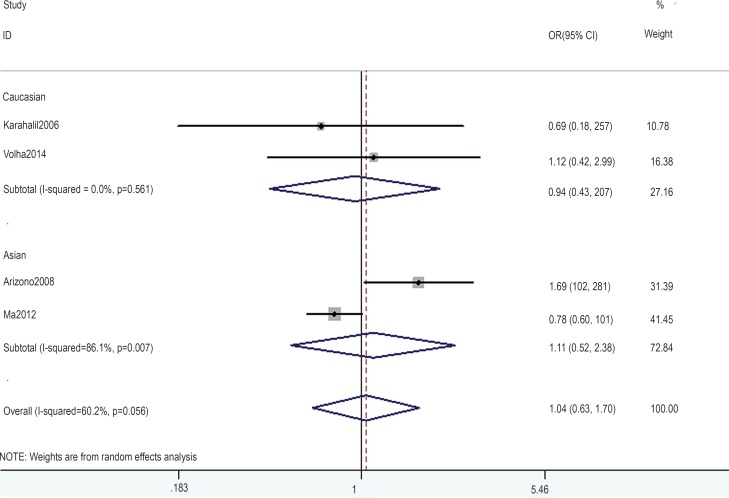
Overall meta-analysis and subgroup analysis by ethnicity for GG genotype versus (GC+CC) genotype in the smoker population.

**Figure 11:**
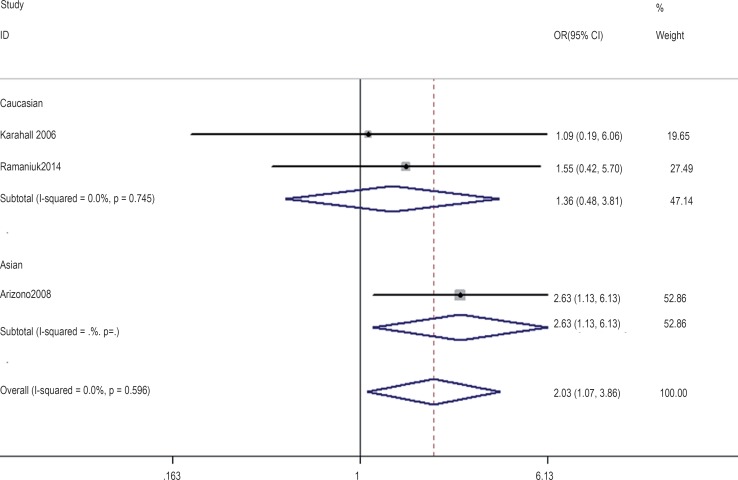
Overall meta-analysis and subgroup analysis by ethnicity for GG genotype versus CC genotype in the non-smoker population.

**Figure 12:**
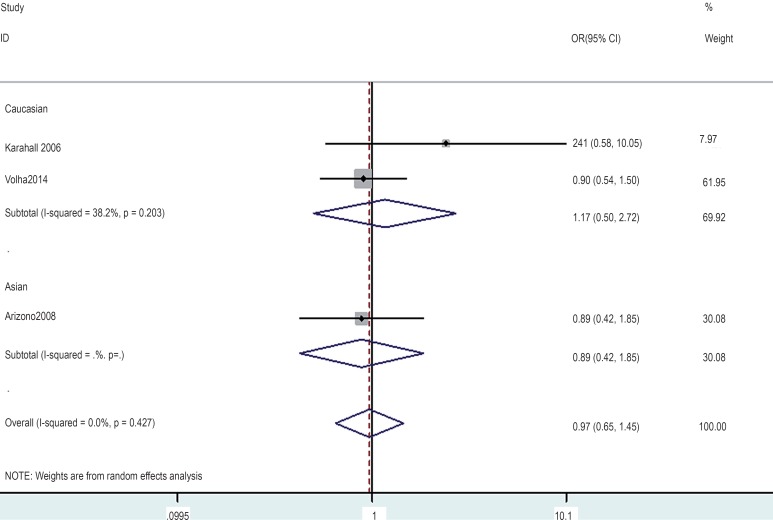
Overall meta-analysis and subgroup analysis by ethnicity for GC genotype versus CC genotype in the non-smoker population.

**Figure 13:**
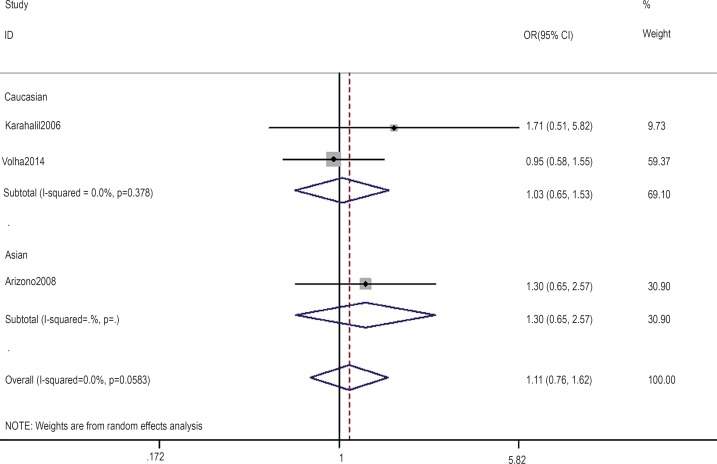
Overall meta-analysis and subgroup analysis by ethnicity for (GG+GC) genotype versus CC genotype in the non-smoker population.

**Figure 14:**
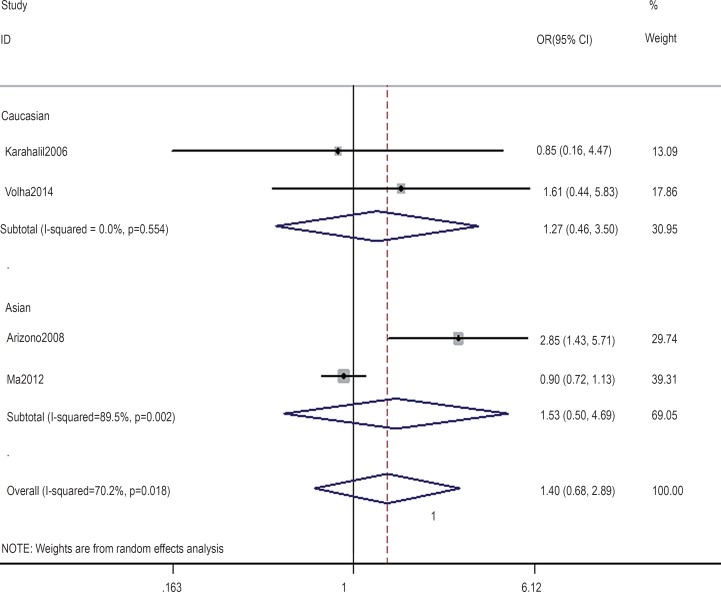
Overall meta-analysis and subgroup analysis by ethnicity for GG genotype versus (GC+CC) genotype in the non-smoker population.

In the gene-by-environment interaction analyses, there were no significant associations between the hOGG1 326Cys polymorphism and bladder cancer in different models for the smoker population, and no ethnicity difference. In the non-smoker population, individuals carrying the hOGG1 Cys326Cys genotype have increased risk for bladder cancer compared to those carrying the hOGG1 Ser326Ser genotype (OR: 2.03, 95%CI: 1.07–3.86; p=0.031). However, this result could not be found in Caucasian population in the codominant model of GG vs. CC for the non-smoker population by subgroup analysis (OR: 1.36, 95% CI: 0.48–3.81; p=0.562), and no significant association was found in the other model for the non-smoker population. The results are shown in Figures 7–14.

### Test of Heterogeneity, Sensitivity Analyses, and Publication Bias

**Figure 15:**
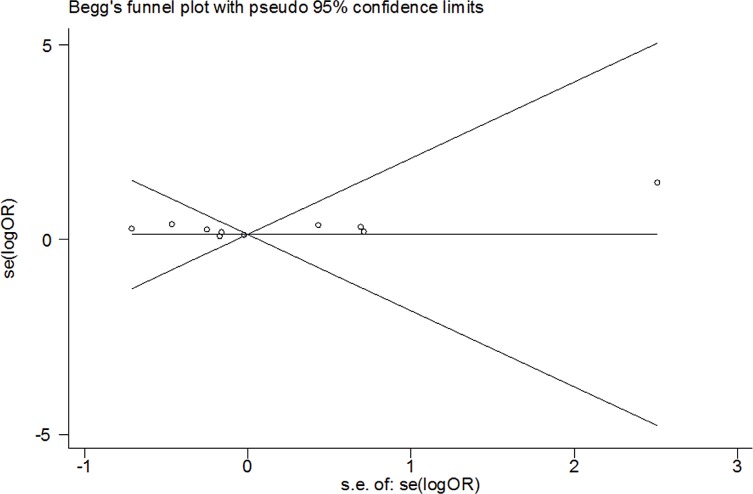
Begg's funnel plots for publication bias in the meta-analysis.

**Figure 16:**
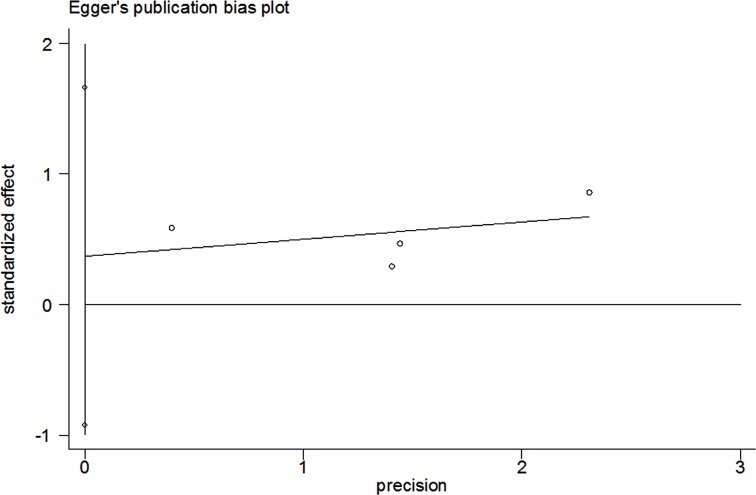
Egger's funnel plots for publication bias in the meta-analysis.

There was significant heterogeneity between studies in some comparisons. Because of the genotype distribution in the control groups deviating from HWE, the study by Karahalil was excluded to test the sensitivity of the meta-analysis, and no substantial change was observed in the corresponding pooled OR was not altered. Funnel plot showed significant symmetry and an absence of publication bias in this meta-analysis. The results are shown in Figures 15, 16.

## DISCUSSION

DNA damage response pathways mainly defend DNA damage, mutations and ultimately malignant caused by various factors, and base excision repair plays an important role in the repair of modified and lost bases (25). As a key gene in the BER pathway (26), the hOGG1 gene polymorphism is closely related to the occurrence of bladder cancer. Many scholars had researched the association between the hOGG1 gene polymorphism and the occurrence of bladder cancer, but the results were inconsistent (19, 27, 28). We carried out this meta-analysis to pool the results from these conflicting findings. Cigarette smoking is considered the main risk factor for bladder cancer. Strope (29) think when smokers with bladder cancer quit after the diagnosis, their risk of recurrence was reduced by 30 percent compared with those who continued to smoke. So, gene-by-environment interaction analyses were also conducted in this meta-analysis.

A total of 10 case-control studies were included in this meta-analysis. The study subjects of 5 studies were Asians, and the other half were Caucasian. So, we carried out the subgroup analyses by race. The results demonstrated that no significant associations were observed between the hOGG1 gene polymorphism and the occurrence of bladder cancer in all models, and this result was no different between Asians and Caucasians (all to). Additionally, subgroup analyses indicated for smokers, there were also no correlations between the hOGG1 gene polymorphism and bladder cancer in all model and this irrelevance had no racial differences. However, the genotype GG is a risk factor to bladder cancer for non-smokers (OR: 2.03, 95% CI: 1.07–3.86; p=0.031), especially in Asians. But no associations were observed in Caucasian and other models. The previous meta-analysis showed the hOGG1 gene polymorphism increases the occurrence of bladder cancer for most all non-smokers (16), but our result point this risk only be found in the non-smoking Asian with GG genotype by pooling the results of much more eligible studies. The detailed mechanism of the hOGG1 gene polymorphism and bladder cancer is unclear and the latest research shows the hOGG1 gene polymorphisms are negatively correlated with bladder cancer in Chinese women, so more large sample size studies are needed to confirm the role of the hOGG1 gene polymorphism in bladder cancer.

There are some limitations in this study. First, single case-control studies can only support a small test power and often provide false-positive, false-negative, or inconsistent conclusions, and the cases number from eligible studies are relatively small. What's more, some detailed information such as gender and histological type could not be obtained, so we could not proceed to the more in-depth subgroup study. And most of the eligible studies were published by the Asian and Caucasian, various race studies are needed to obtain more convincing results. Third, most of the designs of experiment were hospital-based and some studies genotype distributions among the controls are deviated from the HWE.

In conclusion, our meta-analysis suggests that the hOGG1 gene polymorphisms have no significant association with bladder cancer risk, but specifically increase the susceptibility for non-smoker Asian populations. Considering the limited studies in both overall and subgroup analyses, larger sample size and higher quality studies are needed to make further verification.
